# Evaluating the Impact of Optokinetic Stimulation on Weight Balance and Muscle Strength in Healthy Individuals in Virtual Reality During Squats

**DOI:** 10.7759/cureus.79197

**Published:** 2025-02-18

**Authors:** Junya Komagata, Yuki Komatsu, Atsushi Sugiura, Atsuya Otsuka, Toshihiro Kitama

**Affiliations:** 1 Department of Physical Therapy, Nagoya Women’s University, Nagoya, JPN; 2 Department of Rehabilitation, Kamiina Seikyo Hospital, Kamiina, JPN; 3 Center for Life Science Research, University of Yamanashi, Yamanashi, JPN; 4 Department of Physical Therapy, Health Science University, Fujikawaguchiko, JPN

**Keywords:** electromyography (emg), muscle strength, optokinetic stimulation, postural balance, virtual reality (vr), weight-bearing

## Abstract

Background

Asymmetry in lower limb muscle strength and weight-bearing imbalance, commonly observed among athletes, individuals with unilateral impairments, and stroke survivors, require targeted interventions to correct left-right imbalances. Such corrections are essential for enhancing athletic performance, minimizing injury risks in athletes, and improving gait functionality and daily activity efficiency in patients. This study explored, in healthy participants, the potential of combining squat training with optokinetic stimulation (OKS) in an immersive environment to improve left-right balance and address asymmetries in muscle activity and weight distribution.

Methodology

OKS was delivered using a random dot pattern rotating around either the longitudinal axis (horizontal OKS (HOKS)) or the frontal axis (torsional OKS (TOKS)) during squats. Weight-bearing was evaluated by analyzing the center of pressure (CoP) position and foot pressure (FP). Electromyography (EMG) activity was recorded from the following four leg muscles: the vastus lateralis, vastus medialis, semitendinosus, and biceps femoris.

Results

Both HOKS and TOKS increased the CoP sway during squats by 38.6% and 58.6%, respectively, compared to the control. However, only TOKS caused a significant weight-bearing shift toward the stimulus side. During TOKS, FP significantly increased by 10.9% on the stimulus side and decreased by 8.9% on the non-stimulus side, reinforcing the weight-bearing shift. Additionally, EMG activity was significantly elevated by 3.4% on the stimulus side during TOKS, both during the flexion and extension phases. Safety was confirmed during all measurements.

Conclusions

These findings indicate that TOKS induces a shift in weight-bearing and enhances muscle activity, highlighting its potential as a therapeutic intervention for correcting asymmetrical patterns in muscle activity and weight distribution.

## Introduction

In the field of sports, asymmetry in weight-bearing balance and lower limb strength are critical factors that significantly affect athletic performance and increase injury risks. Sports such as gymnastics, weightlifting, rowing, swimming, skating, and cross-country skiing inherently involve symmetrical movements and exercises. In contrast, sports that require asymmetrical movements, such as baseball, soccer, tennis, and javelin, often incorporate uneven strength training. This includes the so-called unilateral exercises, rotational exercises, or uneven loading activities such as medicine ball throws. However, in such sports, excessive loading on a single lower limb can further increase injury risks [1]. Notably, athletes with more than 15% strength asymmetry between limbs are at a markedly higher risk of injury compared to those with smaller imbalances [2]. Therefore, addressing left-right muscle strength imbalances is essential for both injury prevention and performance enhancement in athletes.

In the field of rehabilitation, patients with hemiplegic stroke are often characterized by a standing posture wherein they bear weight predominantly on the non-paretic side, resulting in an asymmetrical balance. These patients tend to exhibit greater postural sway of the center of pressure (CoP) during both steady standing and gait, which increases their risk of falls. Reducing lateral asymmetry by encouraging weight-bearing on the paretic side could be crucial for improving both walking ability and fall prevention. Moreover, among patients with injuries such as anterior cruciate ligament (ACL) injury or total knee arthroplasty (TKA), the issue of weight-bearing asymmetry, which is closely related to muscle weakness on the affected side, commonly occurs during both preoperative and postoperative periods. During recovery from an ACL injury, muscle weakness on the affected side frequently arises, adversely impacting weight-bearing balance and stability in both sports performance and daily activities [3].

In the case of patients with TKA, they often load the healthy knee more owing to pain and a limited range of motion in the affected knee. Even after TKA surgery, as the patient initially adapts to the prosthetic knee, they may tend to rely on their healthy side. Therefore, a gradual increase in weight-bearing on the operated side, along with muscle strengthening and recovery of the range of motion, is required as part of the rehabilitation process during recovery.

Muscle strength training is widely practiced in fitness programs and rehabilitation, playing a vital role in enhancing physical performance and promoting independence in daily activities [4,5]. Among these exercises, squats, a compound movement engaging the hip, knee, and ankle joints, are particularly effective for improving lower limb strength and are extensively utilized to enhance balance and postural stability [6]. Such muscle training benefits healthy individuals and supports patients undergoing rehabilitation by promoting functional recovery and enhancing quality of life. This approach is particularly beneficial for patients with conditions such as stroke, ACL injuries, and postoperative TKA. To effectively address issues such as weight-bearing imbalance, instability, and muscle weakness, neuromuscular loading techniques that incorporate balance and proprioceptive exercises, such as single-legged squats performed on balance pads or with a BOSU balance trainer, are highly recommended. Similarly, in patients recovering from unilateral TKA, the operated limb often demonstrates reduced weight-bearing capacity and quadriceps strength compared to the non-operated limb. This disparity is linked to asymmetrical lower limb movement patterns during functional tasks, such as sit-to-stand transitions [7].

In stroke survivors, motor paralysis and related impairments reduce weight-bearing on the paretic side, leading to disuse atrophy that further exacerbates muscle weakness. This muscle weakness contributes to gait abnormalities and difficulties in daily activities [8]. Rehabilitation aimed at restoring unilateral muscle strength is essential for promoting functional recovery and improving quality of life. Electrical muscle stimulation has demonstrated efficacy in strengthening lower limb muscles and is commonly used in rehabilitating paretic limbs. Conversely, physical exercises such as squats are effective not only for building muscle strength but also for improving balance, movement control, and muscular coordination [9], making them particularly beneficial for individuals recovering from TKA or stroke.

Previously, methods utilizing optokinetic stimulation (OKS) projected onto a large visual scene have been proposed to improve asymmetrical weight-bearing by shifting balance toward the affected side [10,11]. Recently, the application of virtual reality (VR) technology has expanded not only in the fields of gaming and entertainment but also in medicine and medical education, enabling cost-effective and user-friendly visual presentations that encompass the entire field of view without requiring large-scale equipment. Komagata et al. [12] demonstrated three key findings using OKS delivered via a head-mounted display (HMD) in an immersive VR environment. First, they observed a weight-bearing shift, evidenced by a shift in the mean position of the CoP toward the direction of OKS and an increase in the distribution area extending toward the side of OKS. Second, they observed an increase in foot sole pressure on the stimulated side. Third, they reported a lateral shift in the walking trajectory and an increase in the stance period during self-paced gait. Yang et al. [13] demonstrated that gait training in a VR environment is effective for stroke survivors, reducing lateral asymmetry and improving outdoor walking abilities. Consequently, VR is considered a promising approach for addressing lateral asymmetry. However, whether the effects of VR techniques are equally pronounced during dynamic, active movements, such as squats, has not been investigated to date. Therefore, we hypothesized that integrating VR techniques with the commonly used squat exercise would induce a unilateral weight-bearing shift, thereby facilitating the selective training of one side. Additionally, we aimed to evaluate the effects of VR-enhanced squat training on weight-bearing and muscle activity in the lower limb on the side corresponding to the direction of the OKS.

## Materials and methods

Participants

A prior sample size calculation using G*Power 3.1 (Heinrich-Heine-University Düsseldorf) indicated that 22 participants were required for this study to achieve 80% power (α = 0.05, effect size = 0.50) for examining the primary outcome. Consequently, 23 student participants (16 males, seven females; age = 21.1 ± 0.8 years; mass = 55.8 ± 7.8 kg; height = 167.8 ± 6.0 cm) were recruited from the Health Science University.

The co-authors visited the university to conduct data collection. The experimental setup, including HMD and electromyography (EMG) sensors, was prepared in advance and brought to the data collection site for use during the experiment. The collected data were shared online for analysis. The participant inclusion criteria were as follows: (1) ability to perform activities of daily living independently without the use of assistive devices, and (2) normal or corrected-to-normal vision. The participant exclusion criteria were as follows: (1) a history of numbness or sensory disturbances in the lower limbs; (2) severe visual impairments such as amblyopia; and (3) the presence of pain in the hip or knee joints.

This study was conducted in accordance with the guidelines of the Declaration of Helsinki and received approval from the Research Ethics Committee of Health Science University (approval ID: R3-004) and the Ethics Committee of Kamiina Seikyo Hospital (approval ID: 20230012). All enrolled participants provided written informed consent.

Procedures

Before the recording session, participants practiced until they became accustomed to performing squats with proper flexion angles and rhythm. Participants, wearing insole sensors (BodiTrak Insole system; VISTA MEDICAL Co., Ltd., Winnipeg, MB, Canada; sampling rate: 30.3 Hz), stood with their feet shoulder-width apart on a stabilometric platform (Gravicoder GP-5000; ANIMA Co., Ltd., Tokyo, Japan; sampling rate: 100 Hz). They crossed their arms in front of their chest and performed shallow squats with a knee flexion of 60° (knee angle of 120°), monitored using an electronic goniometer (MLTS700, AD Instruments NZ Limited, Dunedin, New Zealand). They performed five repetitions of the squats, each comprising a 2.0-second downward (flexion) and a 2.0-second upward (extension) motion phase, followed by a 2.0-second resting period, standardized using a metronome. Routinely, before the experiment, the equipment for the stabilometric platform and EMG sensors was calibrated according to the instructions for use, and the insole foot-pressure sensors were calibrated using a dedicated device. Ambient conditions, including brightness and room temperature, were consistently controlled and maintained. The recording session began after participants wore the HMD, EMG, and an electronic goniometer. During the experiment, one experimenter stood beside the participant, and handrails or chairs were placed in front of the participant to address safety concerns and eliminate the risks of dizziness or falls. Under each OKS condition, participants completed five repetitions of squats. The CoP was measured using a stabilometric platform, and foot pressure (FP) was measured using an insole sensor. EMG activity was recorded using wireless surface EMG sensors (Delsys Trigno; Delsys Inc., Natick, MA, USA; sampling rate: 2000 Hz) from four leg muscles, namely, vastus lateralis (VL), vastus medialis (VM), semitendinosus (ST), and biceps femoris (BF). These muscles were selected because they are generally active during squatting and represent various muscle depths, including those activated at the shallow angles used in this study. EMG recordings focused on the dominant leg, primarily owing to the system’s limitation in handling simultaneous recordings. Additionally, this study aimed to improve weight-bearing and muscle strength on a specific side, such as the operated side in postoperative TKA patients or the paretic side in stroke survivors. The participant’s dominant leg was determined based on their response to the question of which leg they used to kick a ball. For example, participants who reported using their right leg were classified as right-dominant, while those who reported using their left leg were classified as left-dominant. The placement of sensor electrodes and the procedures followed the guidelines outlined in a previous study [14].

Stimulation, recording, and analysis

The squat test was conducted in an immersive VR environment projected using a stereoscopic HMD (Oculus Rift 2, LLC, Irvine, CA, USA). The experiments were conducted in a dimly lit room to eliminate the effects of light gaps around the edges of the HMD. The virtual imaging system was developed using Unity3D (Unity Technologies, San Francisco, CA, USA) and operated on a desktop PC (ENVY; Hewlett Packard Co., Palo Alto, CA, USA). A total of 3,000 small white virtual spheres, each measuring 25 cm in diameter, were randomly distributed on the inner black surface of a virtual sphere with a 16 m radius from the center of the participant’s eyes. For OKS, after a 10-second wait in a black virtual environment with no visual stimuli, the entire three-dimensional (3D) field with small spheres was rotated around either the participant’s longitudinal axis (horizontal OKS: HOKS) or frontal axis (torsional OKS: TOKS). The stimulus velocity was set at 40°/second as the optimal value for inducing the most effective shift in the CoP, based on a previous study [12]. The stimulus direction was consistently set toward each participant’s dominant leg. For right-leg-dominant participants, this meant using rightward rotation for HOKS and clockwise rotation for TOKS. The control condition, referred to as stationary OKS (SOKS), featured a stationary visual field displaying the same optokinetic pattern, but without any movement. The participants performed squats under these three OKS conditions in a randomized order (randomized block design). Each block included all three stimuli in a randomized order that was generated using the randperm function of MATLAB R2022b (MathWorks, Inc., Natick, MA, USA). This process ensured an equal number of presentations for each stimulus while maintaining randomization. A fixed random seed (random number generator) was used to ensure reproducibility. This procedure was implemented to eliminate systematic biases caused by the order of measurements. Furthermore, each participant was given a sufficient rest period between sessions, which was tailored to their specific needs to account for fatigue or fitness levels.

Each squat motion (excluding the resting period) was detected in the offline analysis based on knee-joint angle data from the electronic goniometer. The flexion and extension phases were determined by differentiating the data to obtain the angular velocity using the criterion of the point where the values crossed two standard deviations of the total distribution. For the CoP data, the sway path and mean were evaluated, and the total distance of CoP displacement on the two-dimensional surface of the stabilometric platform was obtained by combining the x-axis (mediolateral axis) and y-axis (anteroposterior axis) components and the average value of the CoP positions of the x-axis and y-axis components, respectively. The mean FP during each squat phase (mean-FP) and the center position of the FP based on the x-axis component of the pressure distribution on the soles of each foot (center-FP) were also evaluated. EMG activities, sampled at 2,000 Hz and bandpass filtered between 20 and 450 Hz, were rectified. The root mean square (RMS) values for each 500-ms window were computed to derive the EMG amplitude index, which was expressed as a percentage of the maximum EMG recorded during a maximal voluntary isometric contraction (MVIC) for each muscle. The averaged values were calculated for each squat phase (EMG-amp). The maximum amplitude of the four muscles during the squat motion was also expressed as a percentage of the MVIC for each muscle. Furthermore, potential changes in motor unit recruitment were examined by analyzing the median frequency obtained using Fourier transform calculations during squatting under each OKS condition. To evaluate changes in weight balance and EMG activity, the averaged CoP position (sway mean), mean-FP, and EMG-amp during HOKS or TOKS were subtracted from the data during SOKS, which were defined as Δ sway mean, Δ FP, and Δ EMG-amp, respectively.

Custom application programs developed in MATLAB were primarily used for data analysis in this study. All statistical analyses were performed using GraphPad Prism version 6f (GraphPad Software, Boston, MA, USA). One-way analysis of variance was chosen for its suitability in comparing means among multiple groups, assuming a normal distribution and homogeneity of variances. Dunnett’s multiple comparison post-hoc test was used to evaluate the effects of OKS on each data parameter by comparing the HOKS and TOKS condition groups with the control group (SOKS). Possible correlations between Δ EMG-amp and Δ sway mean, or Δ FP were evaluated using Pearson’s correlation. For datasets following a normal distribution, outliers were defined as data points with a Z-score greater than ±3, based on the mean and standard deviation of the dataset. For some datasets that did not follow a normal distribution, outliers were identified using the interquartile range (IQR) method, where data points below Q1 − 1.5 × IQR or above Q3 + 1.5 × IQR were considered outliers. Statistical significance was set at p-values <0.05.

## Results

OKS effect on weight balance during squats

**Figure 1:**
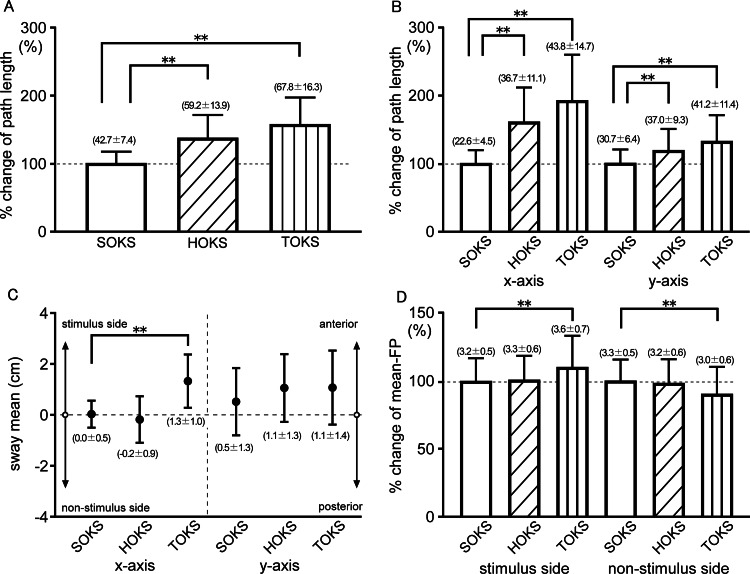
Comparison of the changes in the center of pressure and mean-foot pressure during stationary, horizontal, and torsional optokinetic stimulation. (A) Percent change of total sway path length of the center of pressure by optokinetic stimulation and (B) the percent change of component sway path length in the mediolateral (x-axis) and anteroposterior (y-axis) directions. The numbers in parentheses in (A) and (B) represent the mean ± standard deviation of the actual path length in cm. (C) The mean center of pressure positions in the mediolateral (x-axis) and anteroposterior (y-axis) directions. In the left half of (C), upward indicates the stimulus side, while downward indicates the non-stimulus side. The numbers in parentheses represent the mean ± standard deviation of the actual sway mean values in cm. (D) Percent change of mean-foot pressure on the stimulus and non-stimulus sides for each condition of optokinetic stimulation. *: significant at p < 0.05; **: significant at p < 0.01. CoP: center of pressure; FP: foot pressure; SOKS: stationary optokinetic stimulation; HOKS: horizontal optokinetic stimulation; TOKS: torsional optokinetic stimulation

We examined the effect of OKS on the sway of the CoP position during squat motion. Participants exhibited no signs of distress, including nausea, dizziness, or falls, throughout the recording sessions, thereby confirming the safety of the procedure. During squats under the HOKS and TOKS conditions, the sway path length values were significantly larger than those during SOKS (p < 0.01) (Figure 1A). In addition, both x- and y-axis components showed significant increases compared with those of SOKS (p < 0.01) (Figure 1B), indicating that OKS augmented the sway of the CoP during squats. The x- and y-sway means during squats under SOKS were 0.03 ± 0.52 and 0.52 ± 1.29 cm, respectively, indicating that participants performed squats with an equivalent weight distribution along the mediolateral and anteroposterior axes. During TOKS, the x-sway mean was significantly shifted toward the stimulus side compared to SOKS (1.33 ± 1.02 cm, p < 0.01), while the y-sway mean showed no significant change (1.08 ± 1.42 cm, p = 0.09) (Figure 1C). This indicates that TOKS affects weight balance by shifting it toward the stimulus direction. During HOKS, neither the x-sway mean (-0.18 ± 0.89 cm) nor the y-sway mean (1.06 ± 1.30 cm) showed a clear shift toward the stimulus direction; however, eight out of 23 participants tended to shift their weight balance toward the stimulus. These findings highlight that TOKS significantly influences weight balance, while both TOKS and HOKS increase weight instability.

OKS effect on FP during squats

The change in FP on each foot was analyzed as the CoP position shifted during the squats. During SOKS, the mean-FP of the foot on the stimulus and non-stimulus side showed similar values, indicating an almost even weight distribution along the x-axis (3.2 ± 0.5, 3.3 ± 0.5 N/cm^2^ for the stimulus and non-stimulus side, respectively). During TOKS, the mean-FP on the stimulus side significantly increased by 10.9% compared to that of SOKS (p < 0.01), while the mean-FP on the non-stimulus side significantly decreased by 8.9% (p < 0.01) (Figure 1D). In contrast, mean-FP did not show any significant effect on either the stimulus or non-stimulus side during HOKS. This result suggests that TOKS not only shifted the CoP toward the stimulus side but also increased weight-bearing on the foot on the stimulus side and decreased weight-bearing on the non-stimulus side. The center-FP during SOKS showed no significant difference between the feet on the stimulus and non-stimulus sides (p = 0.8). During TOKS, the center-FP of the foot on the stimulus side shifted toward the stimulus direction by 23.7% compared to that during SOKS (p < 0.05). In the case of HOKS, the center-FP on the stimulus side also tended to shift toward the stimulus direction by 5.1%, but the change was not statistically significant. The center-FP on the non-stimulus side tended to shift toward the stimulus side during TOKS with a shift of 10.3%, but this change was not statistically significant (p = 0.4).

Muscle activity during squats

**Figure 2:**
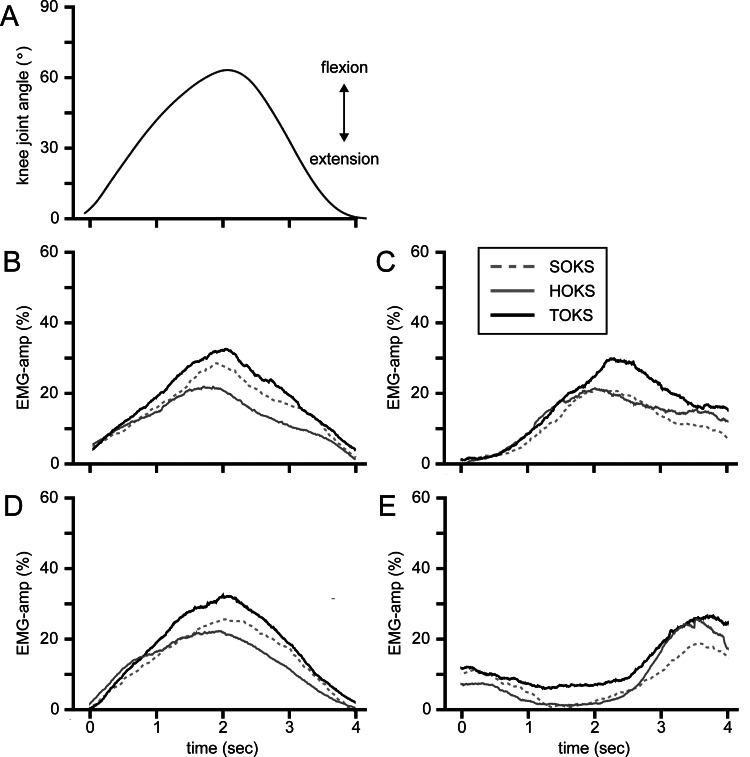
Muscle activity and knee joint angle during squats under optokinetic stimulation. (A) Time-series data of the knee joint angle during squats under optokinetic stimulation (OKS). Upper and lower deviations indicate the flexion and extension phases of the squats, respectively. (B–E) Time-series data of the averaged electromyographic (EMG) activity of four muscles of vastus medialis (B), biceps femoris (C), vastus lateralis (D), and semitendinosus (E) during squats under optokinetic stimulation (OKS), presented on the same time scale as (A). Each trace represents the averaged root mean squared (RMS) values of the filtered and rectified EMG data for each of the four muscles in each participant during squats performed under three OKS conditions: stationary (SOKS, dotted line), horizontal (HOKS, gray solid line), and torsional (TOKS, black solid line) directions. In B-E, the vertical axis represents the EMG amplitude index, expressed as a percentage of the maximum EMG obtained during a maximal voluntary isometric contraction for each muscle. OKS: optokinetic stimulation; EMG: electromyography; SOKS: stationary optokinetic stimulation; HOKS: horizontal optokinetic stimulation; TOKS: torsional optokinetic stimulation

**Figure 3:**
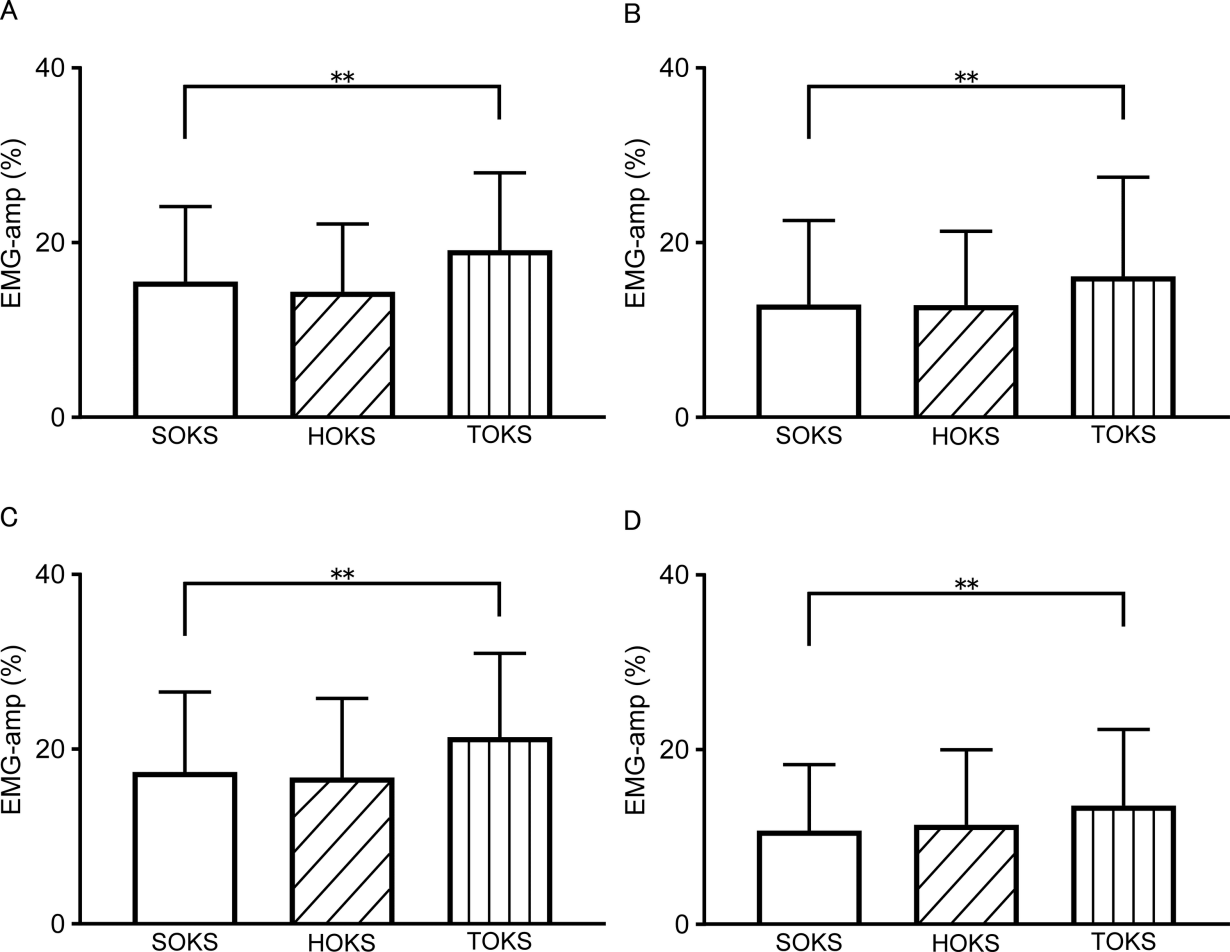
Comparison of electromyographic amplitude index values of four muscles during squats under each optokinetic stimulation condition. Electromyographic (EMG) amplitude of (A) vastus medialis, (B) biceps femoris, (C) vastus lateralis, and (D) semitendinosus, respectively. EMG amplitude index, expressed as a percentage of the maximum EMG obtained during a maximal voluntary isometric contraction for each muscle. **: significant at p < 0.01. EMG: electromyography; SOKS: stationary optokinetic stimulation; HOKS: horizontal optokinetic stimulation; TOKS: torsional optokinetic stimulation; VM: vastus medialis; VL: vastus lateralis; ST: semitendinosus; BF: biceps femoris

**Figure 4:**
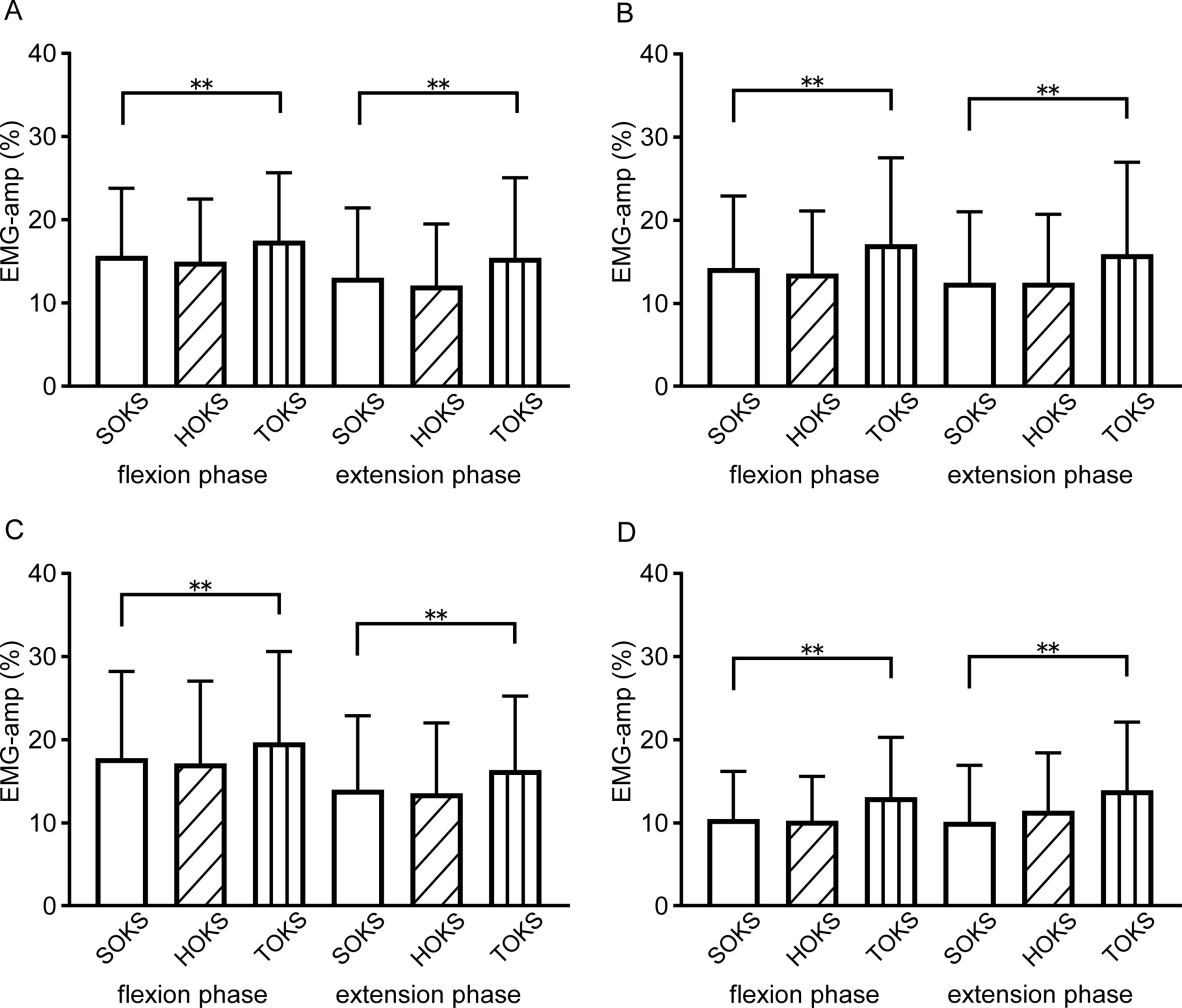
Comparisons of electromyographic amplitude values of each four muscles, separately for the flexion and extension phases. (A-D) Electromyographic amplitude values of (A) vastus medialis, (B) biceps femoris, (C) vastus lateralis, and (D) semitendinosus are shown separately for the flexion and extension phases of squats under optokinetic stimulation. **: significant at p < 0.01. EMG: electromyography; OKS optokinetic stimulation; VM: vastus medialis; VL: vastus lateralis; ST: semitendinosus; BF: biceps femoris

We examined changes in leg muscle activity (EMG-amp) during squats under OKS conditions. The averaged time-series data for all participants during squats under each OKS condition are shown in Figures 2A-2E. During TOKS, all muscles on the stimulus side showed a significant increase of 3.4% (ranging from 2.9% to 4.0%) compared to those during SOKS (p < 0.01) (Figures 3A-3D). However, during HOKS, the EMG-amp of all four muscles did not show any significant changes compared to those during SOKS. Separate analyses of muscle activities during flexion and extension phases of squats revealed significant increases in EMG-amp of all four muscles on the stimulus side during TOKS (p < 0.01) in the flexion phase, with an increase of 2.3% (1.8-2.9%), whereas no significant change (change of -0.5% (-0.7- -0.2%)) was observed during HOKS (p > 0.1) (Figures 4A-4D). Similarly, during the extension phase, EMG-amp of all four muscles showed an increase of 3.0% (ranging from 2.4% to 3.8%) under TOKS (p < 0.01). No significant changes were observed in either the flexion or extension phases during HOKS. Results of the maximum amplitude analysis for each of the four muscles during SOKS were 28.3 ± 14, 28.2 ± 13.9, 26 ± 16.1, and 21.8 ± 13.4％ for VM, VL, BF, and ST, respectively, indicating that the shallow squat performed in this study had mild intensity compared to the MVIC. During TOKS, the index of muscle activity was significantly increased by 4.6, 4.0, 4.9, and 5.5％ for VM, VL, BF, and ST, respectively (p < 0.05), while no clear change was observed during HOKS (p > 0.1).

Next, we examined the median frequency obtained using Fourier transform analysis to investigate possible changes in motor unit recruitment during OKS. The median frequencies during SOKS were 69.1 ± 12, 75.4 ± 20.3, 66.6 ± 15.5, and 74.7 ± 16.9 Hz for VM, VL, BF, and ST, respectively. During TOKS, the median frequency tended to increase across all four muscles; however, a significant increase was observed only in the cases of VM and BF, with increases of 3.6% and 5.3%, respectively, compared to the values during SOKS (p < 0.05). These results suggest slight changes in motor unit recruitment, likely involving the activation of some fast-twitch muscle fibers. In contrast, no noticeable changes were observed in any of the four muscles during the HOKS (p > 0.1).

Relationship between weight-bearing shift and muscle activity change

We separately investigated the effects of OKS on the weight-bearing shift during the flexion and extension phases of squats. The sway means during TOKS significantly shifted during both flexion (0.82 ± 0.88 cm) and extension (1.58 ± 1.50 cm) phases compared with the sway means during SOKS, which were minimal during both flexion (0.01 ± 0.43 cm) and extension (-0.02 ± 0.59 cm) phases (p < 0.01). Similarly, FP during TOKS was significantly higher in both flexion (3.5 ± 0.6 N/cm^2^) and extension (3.5 ± 0.6 N/cm^2^) phases compared with FP during SOKS, which was lower during both flexion (3.3 ± 0.5 N/cm^2^) and extension (3.2 ± 0.5 N/cm^2^) phases (p < 0.01). Conversely, there were no significant changes in sway mean and FP during HOKS compared with those during SOKS in either flexion or extension phases. These results indicate that TOKS induced a significant weight-bearing shift regardless of the squat phase.

Next, we examined the relationship between weight-bearing and muscle activity, conducting separate comparative analyses of muscle activity during the flexion and extension phases of the squats. Correlations between the Δ sway mean, Δ FP, and Δ EMG-amp of all four muscles are shown in Table 1. Δ sway mean exhibited a moderate correlation with all four muscles (p < 0.01). Δ FP was moderately correlated with VM (r = 0.46, p < 0.01) and weakly correlated with BF (r = 0.36, p<0.05). In the flexion phase, Δ sway mean showed a moderate correlation with VM (r = 0.43, p < 0.01); Δ FP was moderately correlated with VM (r = 0.54, p < 0.01) and BF (r = 0.45, p < 0.01) and weakly correlated with ST (r = 0.37, p < 0.05). In the extension phase, the Δ sway mean exhibited a moderate correlation with VM (r = 0.44, p < 0.01), VL (r = 0.36, p < 0.05), and BF (r = 0.39, p < 0.01); Δ FP was moderately correlated with VM (r = 0.63, p < 0.01) and weakly correlated with VL (r = 0.42, p < 0.01) and BF (r = 0.48, p < 0.01). A weak correlation between Δ sway mean and ΔFP was observed throughout the squats (r = 0.39, p < 0.01) and during both flexion and extension phases (r = 0.42 and 0.38 for flexion and extension phases, respectively, p < 0.01). These results suggest that the increases in sway mean and FP induced by OKS were associated with heightened muscle activity. Specifically, during the flexion phase, the activities of VM and BF were more closely associated with the weight-bearing shift, whereas during the extension phase, the activities of VM, VL, and BF showed a stronger association with the weight-bearing shift. Additionally, when examining relationships among muscles, moderate-to-high correlations were observed between the anterior thigh muscles of VM and VL (r=0.81, p < 0.001 for the total squat phase; r = 0.57, p < 0.001 and r = 0.77, p < 0.001 for the flexion and extension phases, respectively); and between the posterior thigh muscles of BF and ST (r = 0.57, p < 0.001 for the total squat phase; r = 0.76, p < 0.001 and r = 0.48, p = 0.001 for the flexion and extension phases, respectively). Furthermore, a moderate correlation was observed between the muscle activities of VM and BF (r = 0.60 and 0.64 during the flexion and extension phases, respectively, p < 0.01).

**Table 1:** Correlations between changes in sway mean, foot pressure, and four muscle amplitudes. Correlation values are presented for the total phase of the squat (top row), and separately for the flexion and extension phases (middle and bottom rows, respectively). Δ sway mean, Δ FP, and Δ EMG-amp were obtained by subtracting the data during stationary optokinetic stimulation from sway mean, mean foot pressure, and electromyographic amplitude during the horizontal or torsional optokinetic stimulation, respectively. *: significant at p < 0.05; **: significant at p < 0.01. VM: vastus medialis; VL: vastus lateralis; BF: biceps femoris; ST: semitendinosus

	Δ EMG-amp							
	VM		VL		BF		ST	
	r	P-value	r	P-value	r	P-value	r	P-value
Total phase								
Δ sway mean	0.58	<0.001**	0.51	<0.001**	0.54	<0.001**	0.56	<0.001**
Δ FP	0.46	0.001**	0.24	0.110	0.36	0.015*	0.21	0.160
Flexion phase								
Δ sway mean	0.43	0.003**	0.10	0.499	0.32	0.030*	0.17	0.258
Δ FP	0.54	<0.001**	0.23	0.117	0.45	0.002**	0.37	0.012*
Extension phase								
Δ sway mean	0.44	0.002**	0.36	0.015*	0.39	0.007**	0.12	0.427
Δ FP	0.63	<0.001**	0.42	0.004**	0.48	0.001**	0.27	0.071

## Discussion

Study strengths

This study investigated whether OKS, presented through an HMD during squats, effectively shifted weight-bearing toward the stimulus direction and increased EMG activity in the leg muscles on the corresponding side. Wearable HMD-VR devices offer a simple, affordable, and user-friendly training tool for both inpatients and individuals post-discharge, with the flexibility to present various types of images, enhancing their scalability. The results demonstrated that OKS through HMD-VR shifted the CoP toward the stimulus direction, increased FP on the stimulus side, and boosted muscle activity on the same side. These findings suggest that VR could be a promising approach for addressing lateral asymmetry such as postoperative TKA patients who experience muscle weakness and reduced weight-bearing on one side. This approach may not only improve muscle strength and balance but also enhance athletic performance and reduce the risk of injury.

Stroke patients often experience a long-term reduction in weight-bearing capacity on the paretic side, which contributes to declines in gait and balance abilities and increases the risk of falls [15]. As a result, there is a growing need for a training tool using an HMD-VR device that can be easily implemented at home. The findings of this study highlight the potential of combining VR technology with strength training as an innovative approach for the rehabilitation of patients with musculoskeletal disorders and stroke. However, in terms of clinical applications, the use of supporting tools such as handrails or chairs and the presence of supporting staff standing beside the participant should be considered essential to address safety concerns and mitigate the risks of dizziness or falls.

Weight-bearing shifts induced by OKS

Displacement of the CoP by OKS has been widely reported. The motion of a large-field visual scene induces a shift in weight-bearing and creates an illusion of self-motion, referred to as “vection,” triggering compensatory responses to maintain an upright posture [16]. The shift in the CoP position or weight-bearing toward the side of the OKS, as demonstrated in this study, aligns with previously reported findings on weight-bearing shifts during static standing in both healthy individuals [12] and stroke survivors [10,11]. Additionally, this study provides evidence of weight balance shifts even during the dynamic movement of squatting. Furthermore, TOKS induces a greater shift in the CoP position compared to HOKS, both in an upright position and during standing-up motions [11,12], as observed in this study.

In patients with hemiplegia following a stroke, decreased weight-bearing on the paretic side is commonly associated with impairments in activities of daily living and walking. Therefore, the observed increase in weight-bearing on the OKS side may help promote greater weight-bearing on the paretic side, which has significant clinical implications. In stroke rehabilitation, various approaches have been utilized to induce weight balance shifts to the affected side, such as training using parallel bars, balance training with a balance board, step training on the affected side supported by the healthy side, and standing action from sitting with gradual weight shifting. While these training methods are recognized to be effective, the approach of combining squats with HMD-VR, as examined in the present study, could provide a more accessible and familiar rehabilitation environment, even for home use after discharge, which could also be scaled to athletic training other than during stroke rehabilitation.

In contrast, other reports have shown the absence of a consistent weight-bearing shift in a specific direction [17]. The impact of OKS on weight balance and vection is more pronounced in the peripheral visual field compared to the central visual field [18]. This phenomenon appears to be caused by the greater amount of visual information derived from the peripheral field owing to its larger size compared to the central field, as suggested by Previc et al. [16]. The HMD device used in this study has a wide visual angle, which creates a highly immersive virtual environment and can effectively induce a weight-bearing shift by presenting the OKS pattern to the entire visual field.

Effect of squats on enhancement of muscle activity

Squats are one of the most common exercises for resistance training to strengthen the lower body muscles, including the muscles of the thigh and surrounding the hip joint [5,6]. Many healthcare and sports workers use squats for rehabilitation training to strengthen these muscles [19]. Considering that many daily activities require the simultaneous and coordinated interaction of multiple muscle groups, the squat is regarded as one of the best exercises for enhancing the quality of life owing to its ability to engage several muscle groups in a single movement. The squatting motion closely resembles many everyday tasks (such as lifting packages and picking up children) and is indirectly related to numerous other chores and hobbies. Continuous squat training has been reported to improve physical functional tests related to activities of daily living [5]. One of our objectives was to integrate the squat motion, combined with the HMD technique, into rehabilitation training for conditions that lead to unilateral lower limb muscle weakness and reduced weight-bearing. The significant increase in EMG activity on the OKS side observed in this study suggests that this approach may be an effective intervention for patients with leg muscle weakness. Furthermore, it has been reported that the degree of post-surgery weight-bearing in TKA patients is primarily influenced by preoperative weight-bearing [20], suggesting that this approach could be applicable not only in postoperative but also in preoperative rehabilitation. This study employed a shallow squat with a knee angle of 120°, as many TKA patients and stroke survivors experience difficulties performing deep squats. Caterisano et al. [21] reported that increasing squat depth enhances gluteus maximus activity. In contrast, the activity of other muscles, including the BF, VM, and VL, remains elevated regardless of squat depth. This indicates that the squat depth employed in this study effectively strengthens these muscles. During TOKS, the four major thigh muscles exhibited a significant increase in activity on the stimulated side, particularly during the flexion phase of the squats. A shift in body balance toward the OKS direction resulted in increased weight-bearing on the corresponding side, which likely triggered elevated muscle activity to support the additional load. Furthermore, such weight balance shifts are positively related to the activity of lower leg muscles, suggesting that the weight balance shift by OKS can consequently improve muscle activity toward the side of the OKS, although this effect is mild. One might argue that the method used in this study does not appear to be particularly effective in addressing lower limb muscle weakness owing to the small observed increases in EMG activity. Certainly, squat training with greater weight loading would likely enhance the effects on muscle activity. For efficient muscle hypertrophy and strengthening, EMG amplitude is generally recommended to reach 60-80% of the activity measured during a maximal voluntary isometric contraction [4]. However, the results of Fourier transform analysis suggested potential motor unit recruitment, possibly involving the activation of some fast-twitch muscle fibers. Repetitive exercises, even with low weight loads, could still produce a certain degree of a muscle-strengthening effect. Muscle strength, particularly that of the quadriceps femoris, has been suggested to play a key role in functional recovery for postoperative TKA patients, because the strength of the operated limb tends to decline over a few years, resulting in prolonged functional disability. In such cases, strength training for the operated limb with a gradual increase in loading weight is considered important and appears to be consistent with the features of this method. The anterior thigh muscles, VM and VL, showed significant increases in activity during both the flexion and extension phases of squats. Similarly, the posterior thigh muscles, BF and ST, exhibited increased activity during both the flexion and extension phases of squats. Generally, squat training focuses on the activation of the quadriceps, which exhibit greater muscle activity than the hamstrings. The use of TOKS increased load in both flexion and extension phases, leading to a significant increase in the activity of the quadriceps (VM and VL) in both phases. However, the hamstrings usually show greater muscle activity during the extension phase of regular squats [22]. Compared to control squats, squats performed with a medially aligned leg position (adduction) increased BF activity during the initial phase of flexion and the beginning of the final flexion phase [22]. This suggests that a weight-bearing shift to the side of OKS appeared to move the hip and knee joints on the stimulated side into an adduction position, likely resulting in increased hamstring muscle activity.

There was a positive correlation between the EMG activity of many muscles and the FP or sway mean values, although this correlation was mild in strength. VM and BF showed a moderate positive correlation with the weight-bearing shift during both the extension and flexion phases, while VL showed a correlation only during the extension phase. A significant difference in muscle activity levels was observed between VL and VM, with the superiority of VL during normal squats [23]. However, with the hip joint in an adduction position, VM activity increased more during squats than VL activity [24]. The anterior thigh muscles, VM and VL, showed significant increases in activity during both the flexion and extension phases of squats. In contrast, the posterior thigh muscles, BF and ST, exhibited increased activity only during the flexion phase. Generally, squat training focuses on the activation of the quadriceps, which exhibit greater muscle activity than the hamstrings. The use of TOKS increased load in both flexion and extension phases, leading to a significant increase in the activity of the quadriceps, VM, and VL, in both phases. However, the hamstrings usually show greater muscle activity during the extension phase of regular squats [22]. Compared to control squats, squats performed with a medially aligned leg position (adduction) increased BF activity during the initial phase of flexion and the beginning of the final flexion phase [22]. This suggests that a weight-bearing shift to the side of OKS appeared to move the hip and knee joints on the stimulated side into an adduction position, likely resulting in increased hamstring muscle activity. There was a positive correlation between the EMG activity of many muscles and the FP or sway mean values, although this correlation was mild in strength. VM and BF showed a moderate positive correlation with weight-bearing shift during both the extension and flexion phases, while VL showed a correlation only during the extension phase. A significant difference in muscle activity levels was observed between VL and VM, with the superiority of VL during normal squats [23]. However, with the hip joint in an adduction position, VM activity increased more during squats than VL activity [24].

Static squats on an unstable disk are reported to decrease lower limb muscle activity [25]. However, Nairn et al. [26] reported that dynamic squats on an unstable disk among college students increased the muscle activity of BF, VM, and rectus femoris up to 1.9 times more than that on a stable surface. Buscà et al. [27] also found that dynamic squats on an unstable disk increased the muscle activity of VL, VM, and BF among athletes. Exercise on unstable surfaces enhances neuromuscular control and increases the activity of stabilizing muscles to maintain core stability [27]. Furthermore, previous studies investigating the training effects using disturbances from an unstable support surface suggested that not only the activity of the primary muscles but also that of the antagonist muscles increases, reinforcing the stability of the lower limb posture through increased joint stiffness [28]. The combination of squats with a whole-body vibration was reported to increase muscle activity by 1.4-5.5% in VM, and 0.5-1.7% in BF, respectively, compared to squats without vibration [25]. In this study, TOKS via VR increased EMG-amp by 2.9-4.0% and the maximum amplitude by 4.0-5.5%, indicating that the approach of this study could induce a relatively good enhancing effect on muscle activity. The use of the vibrating device or weight loading would produce a facilitating effect [25,26].

This study used metronome-paced squats to ensure consistency among all participants. A previous study has shown that comparatively fast-paced volitional squats resulted in higher ground reaction force values compared to slow-paced squats with 10-second flexion and 10-second extension phases [29]. A higher-paced volitional squat or the use of weight loading might have a facilitating effect on muscle activity on the side of OKS. However, slow-paced squats, as used in this study, may be beneficial for improving muscular endurance in patients with conditions such as lower back pain, stroke, or postoperative TKA. Other studies have shown that balance training utilizing visual disturbances for six weeks in patients with stroke resulted in a substantial decrease in the risk of falls six months after discharge. This suggests that visual disturbances, along with physical disturbances, are effective in enhancing lower limb control and joint stability [30]. Squat training with visual disturbance of the OKS in an immersive 3D space might be effective for the stabilization of the lower limbs and joints.

Study limitations

This study has some limitations. First, it focused on healthy individuals rather than TKA or stroke patients, which limits the direct applicability of the findings to rehabilitation settings. Additionally, the lack of reported adverse effects during recording sessions may not fully reflect potential safety concerns. Furthermore, long-term follow-up data assessment is necessary to address the limitations of the sustained effects of this study’s approach. Second, our EMG measurement was performed only for the OKS stimulation owing to the system’s limitation in handling simultaneous recordings. One might think that the increased EMG activity observed could occur in the dominant leg as well as in the non-dominant leg, simply owing to an unstable weight balance caused by the limited measurement being conducted only on the dominant leg, as indicated by the increased sway path length. In other words, the increased EMG activity may not be the result of a shift in weight-bearing but rather a consequence of an unstable CoP or sway in the center of gravity. However, our preliminary experiment, which involved only two muscles (VM and BF) on both sides and was conducted with just three subjects, showed a clear increase in muscle activity on the stimulated side but not on the non-stimulated side. This suggests that the increased EMG activity was caused by a weight-bearing shift rather than an increase in unstable CoP sway. Further studies with a larger sample size are needed to reach a definitive conclusion. Third, there may be certain limitations regarding sample size and participant demographics. This study collected data from a narrow population of healthy students, which may introduce potential bias, although the sample size was statistically sufficient. A larger sample size with a broader age range, such as one including middle-aged and elderly individuals as well as young individuals with varying fitness levels, would provide more comprehensive evidence with greater statistical power, thereby enhancing the generalizability of the results before applying this method to clinical patients. Fourth, while the study’s reliance on EMG activity and CoP or FP data provides insight into muscle activation and weight-bearing shifts, it may overlook other relevant factors influencing rehabilitation outcomes. For example, tracking the movement patterns of the center of mass and the displacement of joints in the feet, knees, and hips would clarify how interconnections between these multiple joints contribute to weight-bearing shifts. Additionally, measuring trunk and pelvic movements would provide further insights into how postural balance relates to weight-bearing shifts. These additional analyses, assessed through motion analysis methods such as motion capture, would contribute to a more holistic understanding of weight-bearing dynamics. Further validation of the study results is necessary before clinical implementation.

## Conclusions

This study investigated the effects of wearable HMD-VR OKS on weight-bearing and leg muscle activity during squats in healthy participants. To our knowledge, no research has examined the effect of combining OKS with the dynamic movement of a squat on both weight-bearing and leg muscle activity, specifically focusing on weight-bearing shifts. No participants reported any signs of distress, such as nausea, dizziness, or falls, during any of the recording sessions. In the future, a pilot study should be conducted to validate the safety of HMD-VR OKS’s effects on weight balance and muscle activity for individuals of a broader age range before implementing this approach in patients with TKA or those undergoing post-stroke rehabilitation. Additionally, in clinical applications, the use of supporting tools should be considered essential to address safety concerns and minimize the risks of dizziness or falls. The method of simple OKS via a VR environment could induce a significant shift in weight balance and muscle activity on the stimulated side. Sustained repetition of the approach used in this study is expected to enhance neural plasticity, facilitate motor function recovery, and improve muscle strength and weight balance in both inpatients and individuals after discharge. In addition, it has potential implications not only for rehabilitation in patients after stroke, ACL injury, or TKA but also for healthy elderly individuals experiencing functional difficulties in gait or sit-to-stand movements in their activities of daily living. An approach using the accessible and familiar environment of VR in this study or augmented reality techniques with other visual presentations in an immersive space rather than OKS would have further scalability. When combined with other devices, such as treadmill-type motion support devices, such an approach could be extended to various fields or applications, including rehabilitation practices and training for both athletes and non-athletes in the near future.
